# Supplementary material for: First Evidence of Anti-Steatotic Action of Macrotympanain A1, an Amphibian Skin Peptide from Odorrana macrotympana
Source: Molecules. 2022 Nov 1;27(21):7417. doi: 10.3390/molecules27217417 (PMC9656375; doi:10.3390/molecules27217417)
Supplement: Supplementary file 1 [file molecules-27-07417-s001.zip › molecules-1933648-supplementary.pdf]

Article

First evidence of anti-steatotic action of Macrotympanain A1, an amphibian skin peptide from *Odorrana macrotympana*

Ilaria Demori[1], Zeinab El Rashed[1], Giulia De Negri Atanasio[1], Alice Parodi[2], Enrico Millo[2], Annalisa Salis[2], Andrea Costa[1], Giacomo Rosa[1], Matteo Zanotti Russo[4], Sebastiano Salvidio[1*], Katia Cortese[3] and Elena Grasselli[1*]

Supplementary Materials

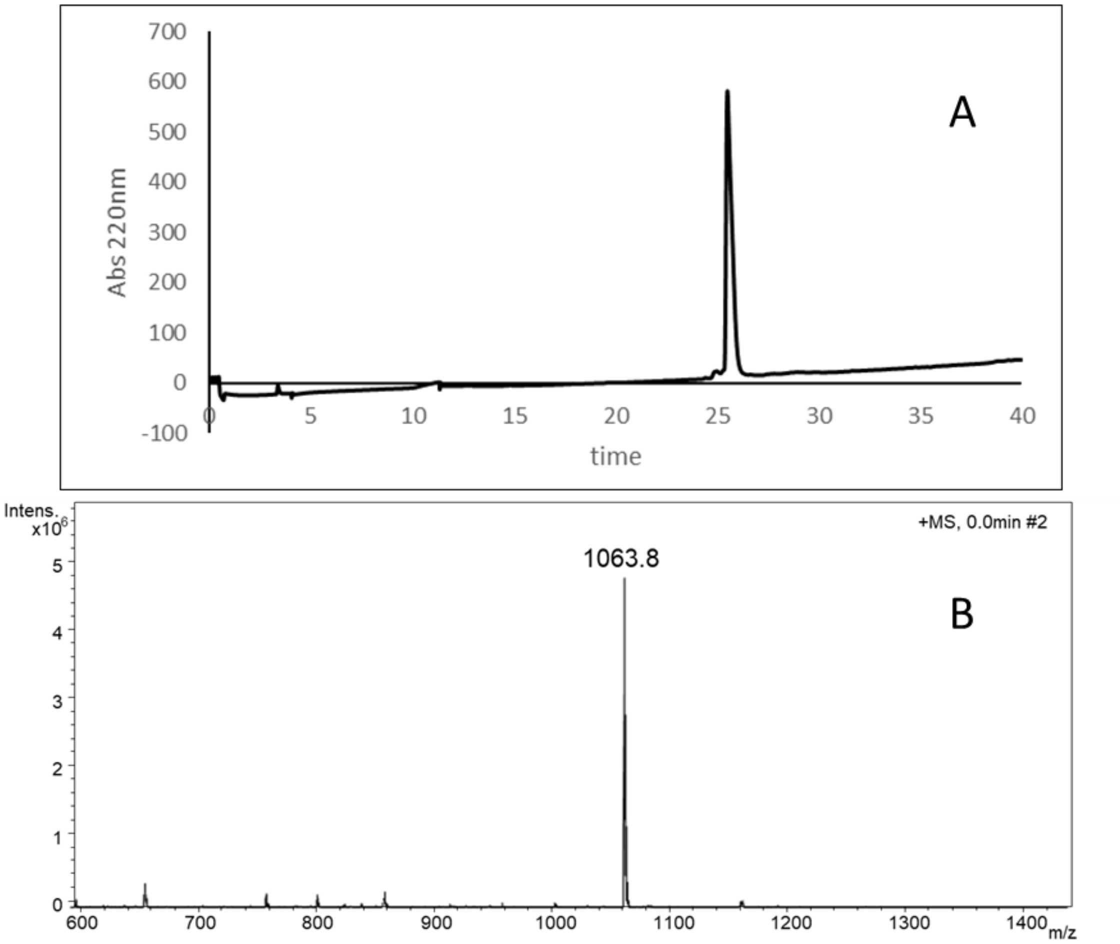

Figure S1: Analysis of purified MA1 peptide. (A): Chromatogram at 220nm of purified peptide. (B): MS spectra of collected peak at Rt 25.1 minutes by off-line MS analysis.
